# Cytotoxicity and ER stress–apoptosis gene expression in ZnO nanoparticle exposed THP-1 macrophages: influence of pre-incubation with BSA or palmitic acids complexed to BSA

**DOI:** 10.1039/c8ra02509f

**Published:** 2018-04-24

**Authors:** Yu Gong, Xianqiang Li, Guochao Liao, Yanhuai Ding, Juan Li, Yi Cao

**Affiliations:** Key Laboratory of Environment-Friendly Chemistry and Applications of Ministry Education, Laboratory of Biochemistry, College of Chemistry, Xiangtan University Xiangtan 411105 P. R. China yhding@xtu.edu.cn juanli@xtu.edu.cn; College of Animal Science, Tarim University, Key Laboratory of Tarim Animal Husbandry Science and Technology of Xinjiang Production & Construction Corps Alar 843300 P. R. China; International Institute for Translational Chinese Medicine, Guangzhou University of Chinese Medicine Guangzhou Guangdong 510006 P. R. China

## Abstract

In a biological microenvironment, biological macromolecules could interact with nanoparticles (NPs) and consequently influence the toxicity of NPs. This study investigated the effects of BSA or palmitic acids complexed to BSA (PA–BSA) on the toxicity of ZnO NPs to THP-1 macrophages. Atomic force microscopy showed the increase of NP heights after pre-incubation with BSA or PA–BSA, but PA–BSA more effectively altered the hydrodynamic size and zeta potential of NPs. Pre-incubation with BSA but not PA–BSA alleviated ZnO NP induced cytotoxicity, and transmission electron microscopy confirmed fewer intrastructural changes after exposure to ZnO NPs pre-incubated with BSA. ZnO NP exposure increased intracellular Zn ions but decreased reactive oxygen species (ROS) and release of soluble monocyte chemotactic protein-1 (sMCP-1), whereas pre-incubation with BSA and PA–BSA induced a different pattern of intracellular Zn ions and modestly increased intracellular ROS. The expression of ER stress marker *DDIT3* was only significantly induced after exposure to NPs pre-incubated with PA–BSA, and *CASP12* expression was significantly lower after exposure to NPs pre-incubated with BSA compared to NPs with or without pre-incubation of PA–BSA. In summary, these results showed that pre-incubation with BSA was more effective compared with PA–BSA to alleviate the toxicity of ZnO NPs to THP-1 macrophages, which should be considered for the evaluation of NP toxicity in a biological microenvironment.

## Abstract 2

In a biological microenvironment, biological macromolecules could interact with nanoparticles (NPs) and consequently influence the toxicity of NPs.

## Graphical abstract

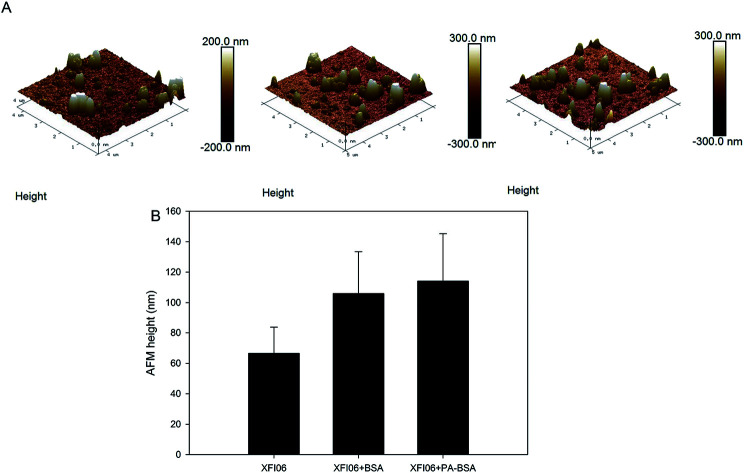

## Introduction

When in a biological microenvironment, nanoparticles (NPs) could rapidly adsorb biological molecules because of the high free surface energy, which can consequently influence the biological effects of NPs. This suggests that it is necessary to consider the interactions between NPs and biological molecules to better evaluate the effects of NPs entering biological microenvironments.^1^ For example, the adsorption of serum proteins to form a protein corona has been extensively studied, and has been convincingly shown to deeply influence the stability and toxicity of NPs in circulation.^2,3^ Still, more studies are needed to investigate the interactions between NPs and biological molecules, especially biological macromolecules other than serum proteins.^4^

Recently, we and others have shown that free fatty acids could be adsorbed onto NPs, which could influence the colloidal stability and biological effects of NPs.^5–7^ However, under *in vivo* conditions a large amount of free fatty acids is complexed to proteins, but relatively few studies investigated the interactions between NPs and free fatty acids complexed to proteins. Lichtenstein *et al.* showed enhanced uptake of Ag NPs into Caco-2 cells if NPs were digested with the main food components, such as carbohydrates, proteins and fatty acids.^8^ Cao *et al.* showed that saturated fatty acids complexed to BSA enhanced the cytotoxicity of ZnO NPs to Caco-2 cells, which was associated with increased mitochondrial reactive oxygen species (ROS) production.^9^

Therefore in this study, we investigated the influences of pre-incubation with palmitic acids complexed to BSA (denoted as PA–BSA) on colloidal aspects and toxicity of ZnO NPs to THP-1 macrophages, and compared the effects with BSA pre-incubated NPs. We focused on the interactions between PA and ZnO NPs because our recent studies showed that the salt of PA could influence the colloidal aspects and toxicity of ZnO NPs,^7,10,11^ but the influences of biological corona formed by PA–BSA on the toxicity of ZnO NPs remain unknown. Atomic force microscope (AFM) was used to visualize the topographic changes of ZnO NPs due to the adsorption of BSA or PA–BSA. The changes of hydrodynamic size, zeta potential and polydispersity index (PDI) were measured to indicate the alteration of colloidal aspects of NPs. Cytotoxicity, intracellular ROS, intracellular Zn ions and release of soluble monocyte chemotactic protein 1 (sMCP-1) were measured in THP-1 macrophages after exposure to ZnO NPs with or without pre-incubation of BSA or PA–BSA. In addition, ultrastructural changes were also observed by transmission electron microscopy (TEM). To indicate the possible mechanisms, the expression of genes associated with ER stress and apoptosis was measured by real time RT-PCR.

## Materials and methods

### Cell culture

The THP-1 monocytes (ATCC) were cultured in RPMI1640 medium (Hyclone, GE Healthcare) supplemented with 10% FCS (GIBCO, South Africa) and 1% P/S solution (Beyotime, Nantong, China) and differentiated into macrophages by using 10 ng mL^−1^ phorbol 12-myristate 13-acetate (PMA; Sigma, St. Louis, MO, USA) as we previously described.^7^

### PA–BSA preparation

PA was complexed to BSA as previously described.^9^ Briefly, PA (purchased from Sigma-Aldrich) was dissolved in EtOH as 200 mM and stored at −20 °C before use. On the day of use, PA was dissolved in 10% BSA as 4 mM. After incubation at 37 °C for about 30 min, PA–BSA was further used for experiments.

### ZnO NP preparation

ZnO NPs (code XFI06; 20 nm) were purchased from Nanjing XFNANO Materials Tech Co., Ltd and have been thoroughly characterized as we reported elsewhere.^12^ The X-ray diffractograms (XRD) indicated the hexagonal phase of NPs with an average size of 22.3 nm. The BET surface area was measured as 19.072 m^2^ g^−1^. The hydrodynamic size and zeta potential of 16 μg mL^−1^ XFI06 suspended in water were measured as 234.4 ± 3.5 nm and −15.5 ± 0.3 mV, respectively.

To make the suspension of ZnO NPs, 1.28 mg mL^−1^ NPs were suspended in MilliQ water containing 1 mg mL^−1^ BSA (Sigma-Aldrich, USA) and sonicated twice for 8 min with continuous cooling on ice by an ultrasonic processor FS-250N (20% amplitude; Shanghai Shengxi, China). After sonication, the NPs were diluted as 40 μg mL^−1^, 160 μg mL^−1^ or 640 μg mL^−1^ in MilliQ water, 5% BSA or 2 mM PA–BSA. After incubated for 30 min, the NPs were diluted ten time in serum free medium to expose cells. Control cells were exposed to equal amount of vehicles in serum free medium.

### *In situ* atomic force microscope (AFM)

AFM was used to visualize the changes of topography of XFI06 due to the incubation with BSA or PA–BSA. Briefly, the XFI06 suspensions were prepared as 640 μg mL^−1^ and incubated with 5% BSA or 2 mM FFA for at least 30 min. After incubation, the suspensions were diluted as 64 μg mL^−1^ in water, 0.5% BSA or 200 μM PA–PBSA. *In situ* AFM experiments were used to investigate the topography on a Bruker MultiMode 8 by using Peakforce mode at room temperature as we described elsewhere. A total of 50 randomly selected NPs was measured in each sample to indicate the changes of AFM heights.

### Hydrodynamic size and zeta potential distribution

The stock solutions of different XFI06 were prepared as indicated above, and diluted as 64 μg mL^−1^ in MilliQ water with or without the presence of 0.5% BSA or 200 μM PA–BSA. The hydrodynamic size and zeta potential distribution as well as polydispersity index (PDI) were analyzed by using Zetasizer nano ZS90 (Malvern, Amesbury, UK). All the samples were measured in triplicate, and mean ± SD was calculated.

### CCK-8 and neutral red uptake assays

The cytotoxicity was assessed by CCK-8 (cell counting kit-8) and neutral red uptake assays by using commercial kits according to the manufacturer's instructions (Beyotime, Nantong, China). Briefly, 2.4 × 10^5^ per well THP-1 macrophages were seeded in 24-well plates and exposed to 0 μg mL^−1^, 4 μg mL^−1^, 16 μg mL^−1^ or 64 μg mL^−1^ XFI06 with or without the presence of 0.5% BSA or 200 μM PA–BSA (in serum free medium). After 6 h exposure, the cells were rinsed once with Hanks' solution, and then the CCK-8 and neutral red uptake assays were done according to manufacturer's instructions. The products were read by an ELISA reader (Synergy HT, BioTek, Woburn, MA, USA).

### Transmission electron microscopy (TEM)

TEM was used to visualize the ultrastructural changes. Briefly, THP-1 macrophages were seeded at 3 × 10^6^ on 60 mm diameter cell culture Petri dishes. The NP suspensions were prepared as indicated above, and the cells were exposed to 64 μg mL^−1^ XFI06 with or without the presence of 0.5% BSA or 200 μM PA–BSA (in serum free medium). After 6 h exposure, the cells were rinsed once and then scratched by using a cell scraper. After centrifuge, the cells were fixed with 2.5% glutaraldehyde in PBS overnight, post-fixed with 1% OsO_4_ for 3 h, dehydrated in a graded series of ethanol, and embedded in epoxy resin (Epon 812). The samples were then sectioned using an ultramicrotome at 70 nm, placed on carbon film supported by copper grids, stained with uranyl acetate and lead citrate, and observed under a TEM (JEM-1230, JEOL Ltd., Tokyo, Japan) operated at 80 kV.

### Intracellular ROS

The intracellular ROS was measured by a fluorescent probe DCFH-DA as previously described.^13^ The fluorescent products were read at excitation 485 ± 20 nm and emission 528 ± 20 nm by an ELISA reader.

### Intracellular Zn ions

The accumulation of intracellular Zn ions in THP-1 macrophages was measured by using a fluorescent probe Zinquin ethyl ester (Sigma-Aldrich, Saint Louis, MO, USA), as we previously described.^7^

### ELISA

The supernatants from CCK-8 or neutral red uptake assays were collected and stored at −20 °C within one month before ELISA analysis. The release of sMCP-1 was determined by an ELISA kit according to manufacturer's instruction (Neobioscience Technology Co., Ltd., Guangzhou, China). The detection limit is 7.8 pg mL^−1^, and the concentrations of sMCP-1 in all the samples were higher than the detection limit.

### Real time RT-PCR

The mRNA level of genes associated with ER stress (*HSPA5*, *DDIT-3*, *XBP-1s*) and apoptosis (*CASP3*, *CASP9*, *CASP12*) were determined by quantitative real time RT-PCR, using GAPDH as internal control. Briefly, 1.2 × 10^5^ per well THP-1 macrophages were seeded on 6-well plates and then exposed to 0 μg mL^−1^ or 64 μg mL^−1^ XFI06 with or without the presence of 0.5% BSA or 200 μM PA–BSA (in serum free medium) for 6 h. After exposure, the cells were rinsed once and total mRNA was extracted using TRI Reagent® following manufacturer's instructions (Sigma-Aldrich, USA). The cDNA was synthesized by using HiFiScript cDNA Synthesis Kit following manufacturer's instructions (Cwbiotech, Beijing, China). The quantitative real-time PCR was done using UltraSYBR Mixture (Cwbiotech, Beijing, China) on PikoReal™ qPCR system (Thermo-Fisher, USA). The primers for each gene were summarized in Table 1. The mRNA levels were expressed as the ratio between the mRNA level of the target genes and the internal control gene using the comparative 2^−ΔCt^ method.

**Table 1:** The forward (F-) and reverse (R-) primers used in this study

Gene names	F-primer	R-primer	Product length
*GAPDH*	ACAGCCTCAAGATCATCAGC	GGTCATGAGTCCTTCCACGAT	104 bp
*DDIT3*	GGAAACAGAGTGGTCATTCCC	GGAAACAGAGTGGTCATTCCC	116 bp
*XBP-1s*	CCGCAGCAGGTGCAGG	GAGTCAATACCGCCAGAATCCA	70 bp
*HSPA5*	GAATTCCTCCTGCTCCTCGT	CAGCATCATTAACCATCCTTTCG	180 bp
*CASP3*	TTTGAGCCTGAGCAGAGACA	GGCAGCATCATCCACACATA	118 bp
*CASP9*	ACAGGCAAGCAGCAAAGTTGTCGA	AGCACCGACATCACCAAATCCTCC	149 bp
*CASP12*	GCTGCCCACCATTGAAAGACT	ATAGCAGATTCATAGACACCAT	119 bp

### Statistics

Unless otherwise stated, the data were expressed as mean ± SD of means of 3–4 independent experiments. Two-way ANOVA followed by Tukey HSD test using R 3.3.3 to compare the difference; *p* value < 0.05 was considered to be statistically significant.

## Results

### The topography of XFI06

**Fig. 1:**
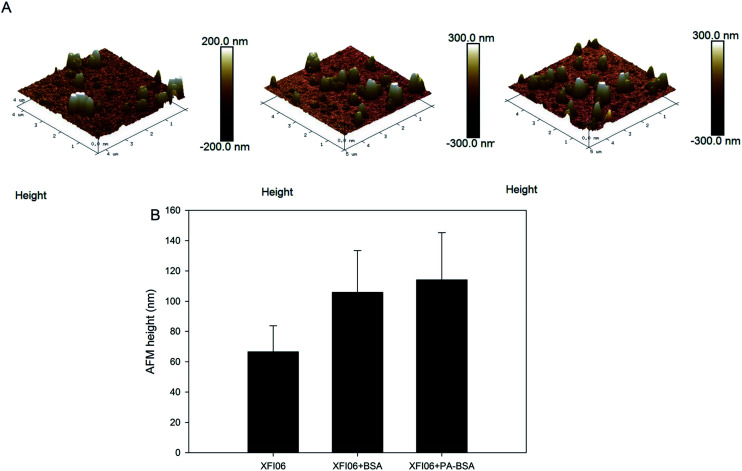
The topography of ZnO NPs (code XFI06) as investigated by AFM. (A) Representative images showing XFI06 (left panel), XFI06 pre-incubated with BSA (middle panel) and XFI06 pre-incubated with PA–BSA (right panel). (B) The changes of AFM height of XFI06 after pre-incubation with BSA or PA–BSA. Data represent mean ± SD of 50 randomly selected NPs.

Fig. 1A shows the topography of NPs investigated by AFM, which indicated the uniform surfaces of XFI06. Pre-incubation with BSA or PA–BSA led to about 70% increase of AFM heights of NPs (Fig. 1B).

### Hydrodynamic size and zeta potential distribution

**Fig. 2:**
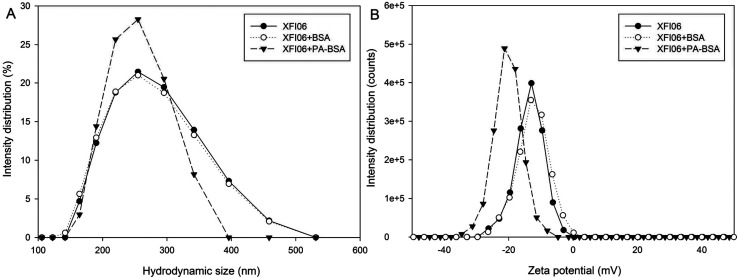
The representative images showing changes of hydrodynamic size (A) and zeta potential distribution (B) of ZnO NPs (code XFI06) with or without pre-incubation of BSA or PA–BSA.

The hydrodynamic size and zeta potential distribution is shown in Fig. 2A and B, respectively. The presence of PA–BSA appeared to be more effective than BSA to shift the hydrodynamic size and zeta potential distribution of XFI06. The average hydrodynamic size of XFI06 was measured as 257.13 ± 4.91 nm, which was increased to 262.60 ± 8.22 nm by BSA and 306.70 ± 13.89 nm by PA–BSA. The average PDI of XFI06 was changed from 0.14 ± 0.04 to 0.17 ± 0.03 by BSA and 0.32 ± 0.04 by PA–BSA. The average zeta potential was measured as −13.47 ± 0.23 mV, which was not significantly changed by BSA (−13.00 ± 0.46 mV) but decreased to −19.63 ± 1.07 mV by PA–BSA.

### Cytotoxicity

**Fig. 3:**
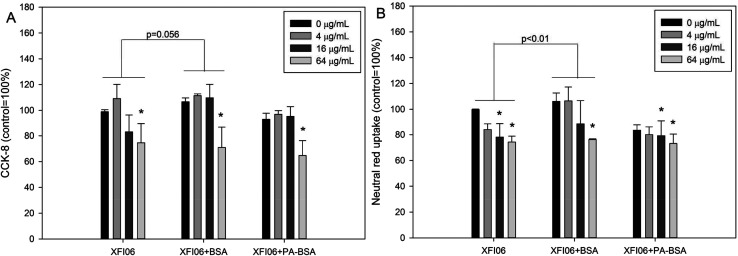
Cytotoxicity of ZnO NPs (code XFI06) with or without pre-incubation with BSA or PA–BSA to THP-1 macrophages. XFI06 was pre-incubated with BSA or PA–BSA for 30 min, and THP-1 macrophages were exposed to various concentrations of XFI06 with or without pre-incubation with BSA or PA–BSA for 6 h. CCK-8 (A) or neutral red uptake assays (B) was used to indicate the cytotoxicity. *p < 0.05, compared with control.

As shown in Fig. 3A, significantly decreased CCK-8 viability was observed in THP-1 macrophages after exposure to 16 μg mL^−1^ XFI06 (*p* < 0.05), 64 μg mL^−1^ XFI06 (*p* < 0.01), 64 μg mL^−1^ XFI06 + BSA (*p* < 0.01), 16 μg mL^−1^ XFI06 + PA–BSA (*p* < 0.05), or 64 μg mL^−1^ XFI06 + PA–BSA (*p* < 0.01). The pre-incubation with BSA or PA–BSA did not significantly the toxicity of XFI06 as assessed by CCK-8 assay, but pre-incubation with BSA led to a relatively higher CCK-8 viability after XFI06 exposure (*p* = 0.056). For neutral red uptake assay (Fig. 3B), significantly decreased neutral red uptake was observed following exposure to 16 μg mL^−1^ XFI06 (*p* < 0.05), 64 μg mL^−1^ XFI06 (*p* < 0.01), 64 μg mL^−1^ XFI06 + BSA (*p* < 0.01), 16 μg mL^−1^ XFI06 + PA–BSA (*p* < 0.01), or 64 μg mL^−1^ XFI06 + PA–BSA (*p* < 0.01). The pre-incubation with BSA (*p* < 0.01), but not PA–BSA (*p* > 0.05), significantly increased neutral red uptake in THP-1 macrophages after XFI06 exposure.

### Ultrastructural changes of THP-1 macrophages

**Fig. 4:**
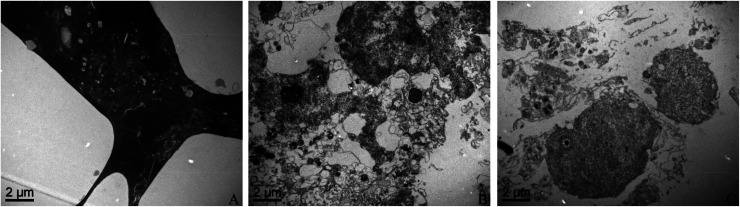
Representative images showing the ultrastructural changes of THP-1 macrophages. XFI06 was pre-incubated with BSA or PA–BSA for 30 min, and THP-1 macrophages were exposed to 64 μg mL^−1^ XFI06 with or without pre-incubation with BSA or PA–BSA for 6 h. TEM was used to observe the morphology of cells after exposure to XFI06 (A), XFI06 pre-incubated with BSA (B) or XFI06 pre-incubated with PA–BSA (C).

TEM was used to indicate the ultrastructural changes of THP-1 macrophages and representative images are shown in Fig. 4. THP-1 macrophages after exposure to XFI06 became relatively dark and showed intracellular vacuolation (Fig. 4A). In contrast, the cells exposed to XFI06 pre-incubated with BSA (Fig. 4B) or PA–BSA (Fig. 4C) were whiter but still exhibited membrane breakage and intracellular vacuolation. The membrane breakage and vacuolation appeared to be more obvious for cells after exposure to XFI06 pre-incubated with PA–BSA compared with cells exposed to XFI06 pre-incubated with BSA.

### Intracellular Zn ions

**Fig. 5:**
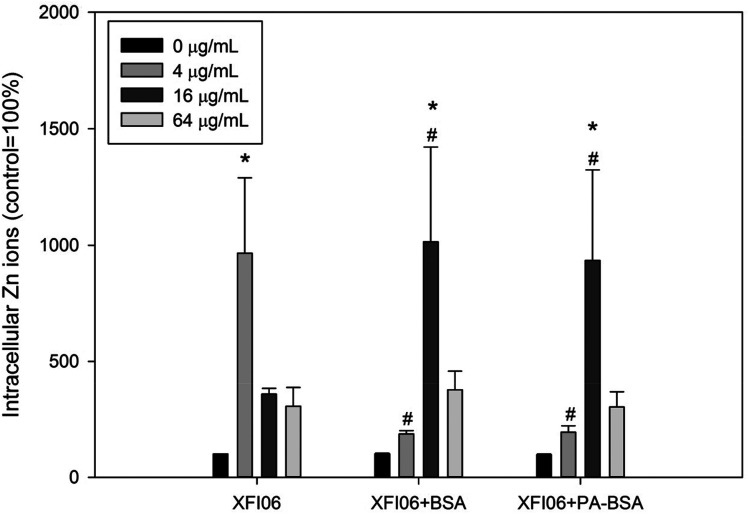
The increase of intracellular Zn ions in THP-1 macrophages after exposure to ZnO NPs (code XFI06) with or without pre-incubation with BSA or PA–BSA. XFI06 was pre-incubated with BSA or PA–BSA for 30 min, and THP-1 macrophages were exposed to various concentrations of XFI06 with or without pre-incubation with BSA or PA–BSA for 6 h. A fluorescent probe was used to measure the intracellular Zn ions. *p < 0.01, compared with control; ^#^*p* < 0.01, compared with the cells exposed to XFI06 at the same concentrations.

Fig. 5 depicts intracellular Zn ions after exposure to XFI06 with or without pre-incubation of BSA or PA–BSA. Exposure to XFI06 increased intracellular Zn ions at all concentrations to different extent, whereas pre-incubation with BSA or PA–BSA changed the pattern of intracellular Zn ion increase. While XFI06 significantly increased intracellular Zn ions at 4 μg mL^−1^ (*p* < 0.01), this concentration was shifted to 16 μg mL^−1^ after pre-incubation with BSA or PA–BSA.

### Intracellular ROS

**Fig. 6:**
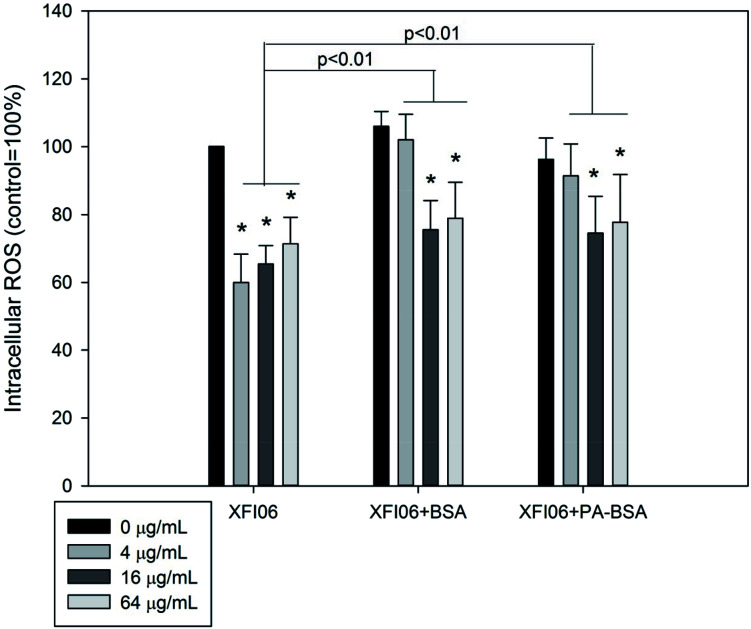
The intracellular ROS in THP-1 macrophages after exposure to ZnO NPs (code XFI06) with or without pre-incubation with BSA or PA–BSA. XFI06 was pre-incubated with BSA or PA–BSA for 30 min, and THP-1 macrophages were exposed to various concentrations of XFI06 with or without pre-incubation with BSA or PA–BSA for 6 h. A fluorescent probe was used to measure the intracellular ROS. *p < 0.01, compared with control.

As shown in Fig. 6, exposure to XFI06 was associated with significantly decreased intracellular ROS at all concentrations (*p* < 0.01). Pre-incubation with BSA or PA–BSA significantly increased intracellular ROS after XFI06 exposure (*p* < 0.01 for both), but still lower than control.

### The release of sMCP-1

**Fig. 7:**
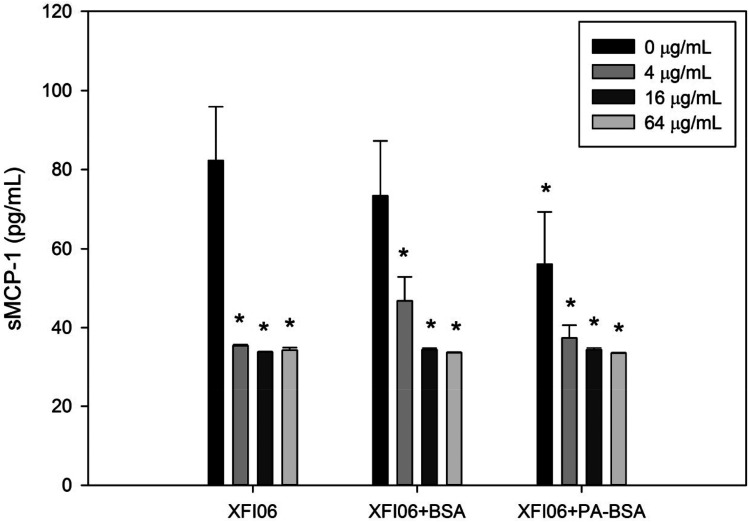
The release of sMCP-1 in THP-1 macrophages after exposure to ZnO NPs (code XFI06) with or without pre-incubation with BSA or PA–BSA. XFI06 was pre-incubated with BSA or PA–BSA for 30 min, and THP-1 macrophages were exposed to various concentrations of XFI06 with or without pre-incubation with BSA or PA–BSA for 6 h. ELISA was used to measure the release of sMCP-1 into the supernatants. *p < 0.01, compared with control.

The release of sMCP-1 was measured by ELISA and result is shown in Fig. 7. The release of sMCP-1 was significantly decreased after exposure to all the concentrations of XFI06 with or without pre-incubation with BSA or PA–BSA (*p* < 0.01).

### The expression of ER stress–apoptosis genes

**Fig. 8:**
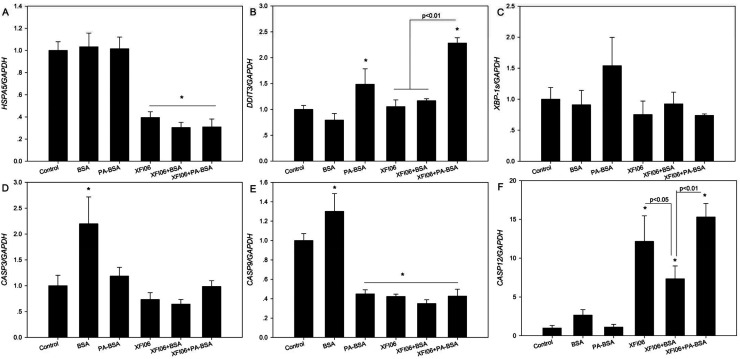
The expression of genes associated with ER stress–apoptosis in THP-1 macrophages after exposure to ZnO NPs (code XFI06) with or without pre-incubation with BSA or PA–BSA. XFI06 was pre-incubated with BSA or PA–BSA for 30 min, and THP-1 macrophages were exposed to XFI06 with or without pre-incubation with BSA or PA–BSA for 6 h. Real-time RT-PCR was used to measure the expression of *HSPA5* (A), *DDIT3* (B), *XBP-1s* (C), *CASP3* (D), *CASP9* (E) and *CASP12* (F). *p < 0.01, compared with control.

Fig. 8 shows the expression of a panel of genes associated with ER stress and apoptosis. For *HSAP5* (Fig. 8A), exposure to XFI06 with or without pre-incubation of BSA or PA–BSA significantly decreased *HSPA5* expression (*p* < 0.01). For *DDIT3* (Fig. 8B), XFI06 with or without pre-incubation of BSA did not significantly affect the expression (*p* > 0.05), whereas XFI06 + PA–BSA significantly increased *DDIT3* expression, which was significantly higher compared with XFI06 or XFI06 + BSA (*p* < 0.01). For *XBP-1s* (Fig. 8C), all the exposure groups did not significantly affect the gene expression (*p* > 0.05). For the gene expression of apoptosis genes, exposure to XFI06 with or without pre-incubation of BSA or PA–BSA did not significantly affect gene expression of *CASP3* (Fig. 8D; *p* > 0.05) but decreased that of *CASP9* (Fig. 8E; *p* < 0.01). For *CASP12* (Fig. 8F), XFI06 with or without pre-incubation of BSA or PA–BSA significantly promoted the gene expression, but the expression of *CASP12* was significantly lower in cells exposed to XFI06 pre-incubated with BSA compared with that in cells exposed to XFI06 (*p* < 0.05) or XFI06 + PA–BSA (*p* < 0.01).

## Discussion

The aim of this study is to investigate the influences of pre-incubation with BSA or PA–BSA on the colloidal aspects and effects of ZnO NPs. The main findings of this study are that both BSA and PA–BSA could influence the AFM height, hydrodynamic size, zeta potential and PDI of ZnO NPs, with PA–BSA being more effective. However, BSA were more effective than PA–BSA to alleviate cytotoxicity and ultrastructural changes of THP-1 macrophages after exposure to ZnO NPs. This could be due to that exposure to ZnO NPs pre-incubated with PA–BSA induced a relatively higher ER stress marker *DDIT3* and apoptosis gene *CASP12* compared with the exposure to ZnO NPs pre-incubated with BSA. Exposure to ZnO NPs increased intracellular Zn ions but decreased ROS and release of sMCP-1, whereas pre-incubation with BSA and PA–BSA induced a different pattern of intracellular Zn ions and modestly increased intracellular ROS.

We first used multiple technologies to indicate the changes of colloidal aspects of NPs due to pre-incubation with BSA or PA–BSA, because it has been suggested that biological molecules could influence the colloidal stability and consequently the toxicity of NPs.^14,15^ The results from AFM showed increased AFM heights of NPs following pre-incubation with both BSA and PA–BSA (Fig. 1). To the best of knowledge, no previous study used AFM to measure the corona formation onto surface of NPs by fatty acids complexed to BSA, but a previous study showed size-dependent binding of NPs to phospholipids by AFM measurement.^16^ It is possible that NPs could adsorb lipids both complexed and not complexed to BSA. In this study we preferred to use AFM rather than TEM to measure the changes of particle sizes due to the adsorption of biological macromolecules, because AFM could allow *in situ* measurement of protein corona, whereas the corona might be changed during the preparation of TEM samples.^3,17^ Recently, we have also used *in situ* AFM to visualize the topographic changes of multi-walled carbon nanotubes after pre-incubation with BSA.^18^ AFM might also be applied for the measurement of interface energy between NPs and matrix as recently shown.^19^ Alternatively, cryo-TEM may overcome the limitations of conventional TEM to preserve protein corona.^20,21^ The results from this study further showed increase of hydrodynamic size and PDI as well as decrease of PDI, which was more obvious for PA–BSA compared with that of BSA (Fig. 2). Recently we also found that the salt of fatty acids could increase the hydrodynamic size and decrease zeta potential of ZnO NPs, but compared with our previous reported data, it appeared that the salt of fatty acids altered these endpoints more effectively compared with PA–BSA.^6,7,10,11^ Nevertheless, AFM height, hydrodynamic size, zeta potential and PDI of ZnO NPs could be changed after pre-incubation with BSA or PA–BSA, which suggested the changes of colloidal aspects of NPs due to the formation of biological protein corona.

We then investigated the cytotoxicity following exposure to ZnO NPs with or without pre-incubation of BSA or PA–BSA. The results from CCK-8 and neutral red uptake assays indicated that pre-incubation with BSA could promote cellular viability of THP-1 macrophages following ZnO NP exposure (Fig. 3). TEM study further confirmed that NPs pre-incubated with BSA induced less ultrastructural of THP-1 macrophages (Fig. 4). This is consistent with previous observations that the presence of protein corona could mitigate the toxicity of NPs.^22,23^ Interestingly, the results from this study also showed that PA–BSA was less effective compared with BSA to reduce the cytotoxicity of ZnO NPs. A previous study showed that PA–BSA enhanced the cytotoxicity of ZnO NPs to Caco-2 cells.^9^ In another study, it was shown that PA–BSA enhanced monocyte adhesion to human endothelial cells after multi-walled carbon nanotube exposure.^24^ We recently suggested that PA might enhance the toxic effects of NPs due to the intrinsic toxicity associated with saturated fatty acids.^11^ This may indicate that the influence of biological protein corona formation on toxicity of NPs is dependent on the types of biological molecules adsorbed onto NPs.

The cytotoxicity of ZnO NPs could be mediated with accumulation of intracellular Zn ions and/or ROS.^25^ In this study, the results showed increased intracellular Zn ions (Fig. 5) but decreased ROS (Fig. 6). In our recent studies, we also showed significantly increased intracellular Zn ions but not intracellular ROS in a variety of cell lines following exposure to the same types of ZnO NPs, which indicated a role of intracellular Zn ions in cytotoxicity of ZnO NPs.^6,11,26–28^ However, although PA–BSA was less protective compared with BSA against ZnO NP exposure, both PA–BSA and BSA altered the pattern of increase of intracellular Zn ions to a similar extent. Recently we found that the salt of PA enhanced the toxicity of ZnO NPs without an influence on NP induced intracellular Zn ions.^7,10^ In addition, it has also been shown that the ER stress inducer promoted the toxicity of ZnO NPs to macrophages without further increase of intracellular Zn ions.^29^ Thus, it is possible that the toxicity of ZnO NPs could be altered by biological molecules without alterations of intracellular Zn ions, and the changes of cytotoxicity of ZnO NPs after pre-incubation with BSA or PA–BSA could be mediated by other mechanisms.

It has been suggested that ZnO NP exposure might modulate inflammatory responses in mammalian cells.^30^ To this end we determined the release of sMCP-1, and results showed that it was significantly reduced after exposure to NPs with or without pre-incubation of BSA or PA–BSA (Fig. 7). Recently we found that ZnO NP exposure significantly reduced IL-6 release from HepG2 cells^12^ or A549 monolayer.^26^ Previous studies also showed that ZnO NPs inhibited inflammatory responses in human mast cells^31^ or macrophages^32^ after LPS challenge. These results in combination with our data suggested that ZnO NPs might inhibit inflammatory responses. Pre-incubation with BSA or PA–BSA did not significantly affect the tendency of sMPC-1 release, which suggested that these biological molecules might not affect the inflammatory potential of ZnO NPs.

To explore the possible mechanisms associated with relatively lower cyto-protective effects of PA–BSA against ZnO NP exposure, we measured key regulators involved in ER stress–apoptosis. ER stress is an adaptive response to accumulation of unfolded proteins which could regulate the apoptosis pathway,^33,34^ and induction of ER stress has been suggested to be correlated with toxicity of NPs.^2,35^ The results from this study showed significantly higher expression of ER stress marker *DDIT3* and apoptosis gene *CASP12* after exposure to NPs pre-incubated with PA–BSA compared with NPs with or without pre-incubation of BSA, whereas NPs with or without pre-incubation of BSA or PA–BSA altered other ER stress–apoptosis gene expression to similar extent (Fig. 8). Since *DDIT3* and *CASP12* are pro-apoptosis genes that could be activated in response to prolonged ER stress,^33–35^ the relatively higher *DDIT3* and *CASP12* expression could be associated with the less cyto-protective effects of PA–BSA. Previous study showed that exposure to ZnO NPs could activate the expression of ER stress genes,^36–38^ here we further showed that the induction of ER stress–apoptosis gene expression is dependent on the interactions between ZnO NPs and biological molecules.

In summary, the results from this study suggested that pre-incubation with BSA or PA–BSA could alter the colloidal aspects of ZnO NPs, but pre-incubation with BSA was more effective than PA–BSA to alleviate ZnO NP induced cytotoxicity and ultrastructural changes of THP-1 macrophages. Exposure to ZnO NPs was associated with increased intracellular Zn ions but decreased ROS and release of sMCP-1, whereas pre-incubation with BSA and PA–BSA altered the pattern of intracellular Zn ions and ROS production to similar extent. Co-exposure to PA–BSA and ZnO NPs induced a relatively higher ER stress marker *DDIT3* and apoptosis gene *CASP12* compared with the exposure of ZnO NPs pre-incubated with BSA, which is likely associated with the changes of toxicity of ZnO NPs. The results from this study suggested complex interactions between NPs and biological macromolecules, which should be considered in future studies when assessing the toxicity of NPs in a biological microenvironment.

## Conflicts of interest

No.

## Supplementary Material
